# Effects of Grape Seed Extract-Modified Etchants on Collagenolytic Activity, Interface Formation, and Bonding Longevity of Adhesive–Dentin Interfaces

**DOI:** 10.3390/ma18112416

**Published:** 2025-05-22

**Authors:** Viviane Hass, Xiaomei Yao, Yong Wang

**Affiliations:** Department of Oral & Craniofacial Sciences, University of Missouri-Kansas City School of Dentistry, Kansas City, MO 64108, USA; hassv@umkc.edu (V.H.);

**Keywords:** acid etching, dentin collagen crosslinking, grape seed extract, polyphenols, collagenase, MMPs, adhesive–dentin interface

## Abstract

This study investigated the effects of acid etching with grape seed extract (GSE)-modified etchants, varying phosphoric acid (PA) concentrations, on endogenous collagenolytic activity of etched dentin, adhesive–dentin (A/D) interfacial formation, and bond strength over time. Three PA concentrations (5%, 10%, and 20%) were combined with 2% GSE (5PA/GSE, 10PA/GSE, and 20PA/GSE) and compared to a control (CT) group using 32% PA gel (3M Universal Scotchbond etchant). Seventy-four caries-free human third molars were sectioned to expose dentin surfaces, which were etched and analyzed. In situ zymography with confocal laser microscopy was used to assess endogenous collagenolytic activity in etched dentin specimens. For A/D interfacial morphology and bond strength, etched dentin was bonded with Adper Single Bond Plus adhesive (3M ESPE) and composite buildup. The interfacial morphology of A/D specimens was evaluated using either Goldner’s trichrome staining under light microscopy after microtomy sectioning or scanning electron microscopy. A/D specimens were stored in either TESCA buffer or collagenase solution and tested immediately (IM) or at multiple time points over one year using the microtensile bond strength (μTBS) test. Data were analyzed by one- or three-way ANOVA followed by Games–Howell or Tukey’s tests (α = 0.05). GSE-modified etchants significantly reduced endogenous collagenolytic activity (*p* < 0.05). Although GSE-modified etchants resulted in thinner A/D interfaces, the bond strength remained unaffected (*p* > 0.05). Bond strength stability was prolonged up to one year with 5PA/GSE and 10PA/GSE (*p* < 0.001), while CT or 20PA/GSE showed significant degradation by 17 weeks (*p* < 0.01). Storage in the more aggressive collagenase solution did not further reduce the bond strength compared to TESCA buffer (*p* = 0.966). Acid etching with GSE-modified etchants effectively inhibits endogenous MMP-mediated collagenolytic activity. At 5% and 10% PA, this approach enhances the stability of the A/D bond strength, offering a promising modification for dentin bonding protocols.

## 1. Introduction

Despite the widespread use of resin composite restorations over the past 50 years, their clinical success is often compromised by limited stability and longevity. More than half of all restorations placed worldwide are replacements of failed restorations, primarily due to secondary caries at the adhesive–dentin (A/D) interface [1,2]. The A/D interface, which bonds composite restorations to tooth structure, relies on the hybrid layer, a structure of demineralized dentin collagen infiltrated by resin monomers, forming a three-dimensional interlocking network [3]. Consequently, the stability of these interfaces is critical for the longevity of composite restorations [3,4].

Despite their crucial role in supporting and maintaining the composite-tooth complex, the A/D bonding interfaces are inevitably subject to biodegradation within the oral environment. Factors such as incomplete adhesive penetration into dentin [5], the low degree of adhesive polymerization [6], and high water sorption leave collagen fibers unprotected, rendering them susceptible to hydrolytic and enzymatic degradation [4]. Furthermore, the acid etching during bonding procedures activates endogenous collagenolytic enzymes, such as matrix metalloproteinases (MMPs), that are further reactivated by the low pH of adhesive systems [7]. In addition to the endogenous MMPs, recent studies suggest that other exogenous enzymes as collagenases, secreted by oral bacteria [8] or inflammatory cells, also contribute significantly to the degradation of dentin collagen and A/D interfaces [9]. As a result, composite restorations are subjected to a complex enzymatic- and bacterial-rich environment that threatens their longevity.

For nearly 15 years, biomodification using collagen crosslinkers has emerged as a promising strategy to enhance dentin collagen properties and prevent enzymatic degradation [10]. Among natural crosslinkers, proanthocyanidins (PACs) from grape seed extract (GSE) stand out due to their high chemical affinity for collagen, providing rapid and effective stabilization within clinically relevant times [11,12] without cytotoxic effects [13,14]. In particular, PACs exhibit potent MMP-inhibitory activity [14,15], making them particularly valuable in strengthening A/D bonding interfaces [11,14]. However, incorporating PACs into bonding procedures presents a challenge. Like other polyphenols, PACs act as radical scavengers, which can interfere with the polymerization of dental adhesives [16,17]. While PACs have demonstrated efficacy as a dentin primer, this approach requires rinsing before adhesive application to prevent polymerization inhibition [17]. This additional step complicates clinical protocols, increasing treatment time, and reducing clinical practicality.

To overcome the limitations associated with separate application steps, recent research has explored the incorporation of PACs into acid etchants, allowing simultaneous collagen biomodification and demineralization during bonding [18,19,20,21]. This approach is promising because PACs interact with collagen via multiple mechanisms, including hydrophobic interactions between collagen pyrrolidine rings and polyphenol phenyl rings [22]. Importantly, these interactions occur independently of pH-sensitive functional groups, ensuring PAC-mediated crosslinking to remain effective even in the acidic environment of etchants [23]. However, phosphoric acid (PA), with its lower molecular weight and higher hydrophilicity, may etch dentin more rapidly than PACs can crosslink collagen. This discrepancy becomes more pronounced at higher acid concentrations, raising concerns about the compatibility of PACs with commercial etchants, which often contain PA concentrations exceeding 32%, as observed by De-Paula et al. (2020) [24]. To mitigate this issue, PAC-modified etchants are typically formulated with PA concentrations below 20% to achieve a balance between effective demineralization and sufficient time for collagen stabilization [21]. A concentration of 10% PA applied for 30 s has been commonly adopted in experimental models, demonstrating favorable bonding performance while allowing adequate PAC–collagen interactions [18,19,23,25]. However, low PA concentrations such as 10% have been reported to increase endogenous proteolytic activity in demineralized dentin [26,27], which contradicts the intended stabilization effect of PAC-modified etchants and therefore warrants investigation. Moreover, the ability of these lower PA concentrations (when combined with collagen crosslinkers) to consistently form structurally sound and durable A/D interfaces remains unexplored.

Thus, the objectives of this study were to evaluate the effects of GSE-modified acid etchants, as a source of PACs, with varying PA concentrations on collagenolytic activity of endogenous MMPs in etched dentin, A/D bonding interface formation, and dentin bonding stability, particularly bond strength over time. To better simulate the enzymatic-rich environment surrounding composite restorations, we also compared the stability of A/D bond strength using a more aggressive bacterial collagenase solution to that using a TESCA buffer solution. The research hypotheses were that the acid etchants would (1) modify collagenolytic activity of endogenous MMPs, (2) influence A/D bonding interfacial formation, and (3) affect dentin bonding stability, specifically bond strength, in response to variations in acid etchant composition, storage conditions, and evaluation time points.

## 2. Materials and Methods

### 2.1. Etchant and Storage Solution Preparation

All the reagents were purchased from Sigma Millipore (St. Louis, MO, USA) unless otherwise specified. Three PAC-containing etchant formulations were prepared by combining GSE powder, deionized water, ethanol, and 85% phosphoric acid, following Liu 2014 [21]. Each formulation consisted of 2% GSE and 20% ethanol, with either 20%, 10%, or 5% phosphoric acid by total weight; deionized water was added to achieve the final concentrations. The pH of each formulation was measured. The GSE used (MegaNatural Gold, Lot # 05592502-01) was provided by Polyphenolics (Madera, CA, USA). A commercial 32% phosphoric acid gel served as the control. Details of the etchant compositions are provided in Table 1.

Two storage solutions were prepared for use in microtensile bond strength testing. TESCA buffer was prepared by dissolving 11.5 g of N tris(hydroxymethyl)methyl-2-aminoethanesulfonic acid, 50 mg of sodium azide, and 53 mg of calcium chloride dihydrate (CaCl_2_·2H_2_O) in deionized water, adjusted to a final volume of 1 L and pH 7.4. A bacterial collagenase solution was also prepared by dissolving Clostridium histolyticum (type I, ≥125 U/mg) at a concentration of 0.1% (*w*/*v*) in TESCA buffer.

### 2.2. Tooth Preparation and Experimental Design

**Figure 1:**
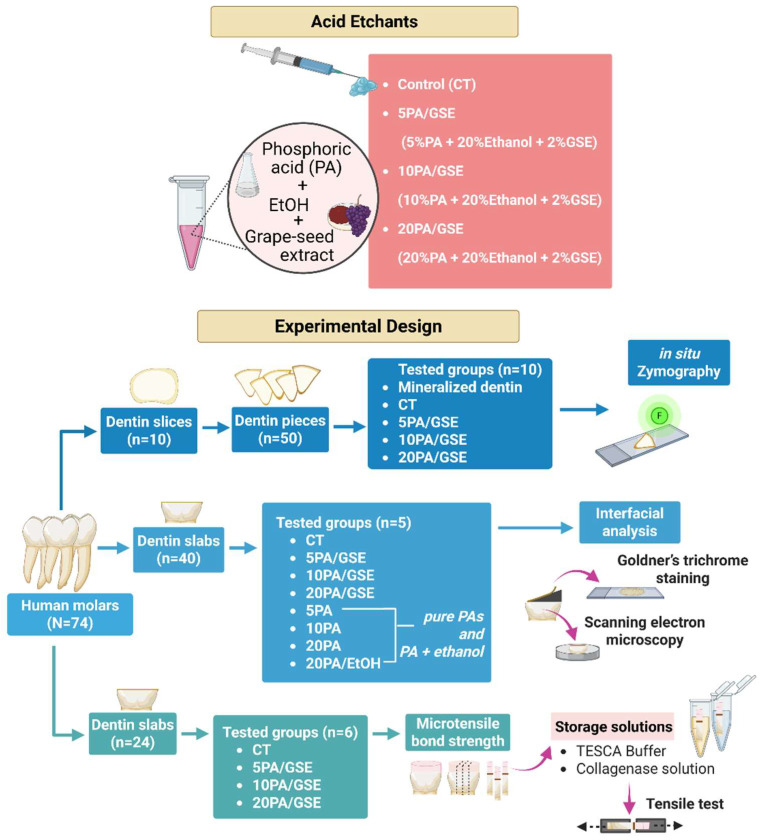
Schematic illustration of the experimental design and characterization flow chart.

Seventy-four caries-free, extracted human third molars were collected as non-human subject research as determined by the University Internal Review Board (12–50 NHSR) and stored in 0.9% (*w*/*v*) phosphate-buffered saline (PBS) containing 0.002% sodium azide at 4 °C for up to one month prior to experimentation. Occlusal enamel and roots were removed using a water-cooled diamond saw (Buehler, Lake Bluff, IL, USA), producing 5 mm thick dentin slabs. A uniform smear layer was created on the exposed dentin surfaces using 600-grit silicon carbide grinding paper (Buehler). The prepared dentin slabs were then allocated to different experimental groups, as outlined in Figure 1.

### 2.3. Collagenolytic Activity of Endogenous MMPs

A total of ten dentin slabs were used to evaluate the collagenolytic activity of endogenous MMPs via in situ zymography. For each slab, a top occlusal slice was sectioned (Buehler) and further cut into five dentin pieces. These pieces were then randomly distributed across four experimental groups based on the acid etchant formulations (Table 1), along with one additional group using mineralized dentin that received no acid etching treatment. This approach ensured consistent dentin substrate across all groups (Mazzoni et al., 2012 [28]). Following DeVito-Moraes (2016) [26] with adaptations, dentin pieces were adhered to microscope slides with cyanoacrylate glue and sequentially polished with wet silicon carbide sandpaper (600, 1200, and 2500 grit) to achieve a final thickness of ~80 μm. Specimens assigned to the etched groups were treated according to their respective acid etchant protocols (Table 1). After rinsing, each etched or mineralized (unetched) dentin specimen was coated with 20 μL of fluorescein-conjugated gelatin solution (E-12055; Molecular Probes, Eugene, OR, USA) and incubated for 72 h in a dark, humid chamber at 37 °C. After incubation, the specimens were rinsed with deionized water and covered with coverslips. Confocal laser scanning microscopy (Leica SP8 Multiphoton, Leica Microsystems, Wetzlar, Germany) was used to image the specimens at 63× magnification using an oil immersion lens and excitation/emission settings of 488/530 nm. For each specimen, three z-stack images (10 μm deep; 1 μm interlayer spacing) were captured from different surface regions. The collagenolytic activity of endogenous MMPs was quantified as the mean fluorescence intensity of green signal using the ImageJ software program version 1.54m (National Institutes of Health, Bethesda, MD, USA) and subjected to a statistical analysis.

### 2.4. Adhesive/Dentin (A/D) Bonding Interfacial Analyses

To better understand the formation of A/D bonding interfaces using GSE-modified acid etchants, additional groups were included comprising etchants formulated with phosphoric acid (PA) alone or PA with ethanol, at the same concentrations as the experimental formulations but without GSE, as detailed in Figure 1. This resulted in eight experimental conditions in total. Forty dentin slabs were randomly distributed among these groups (n = 5 per group) and etched following the same procedures as previously described. Excess moisture was gently removed using absorbent paper (KimWipes, Kimberly-Clark Professional, Roswell, GA, USA). Two consecutive coats of Adper Single Bond Plus adhesive (3M ESPE, St. Paul, MN, USA) were applied: the first layer was air-dried for 5 s, the second layer for 10 s, and the layers were light-cured for 20 s using a curing unit (Dentsply Spectrum 800, Milford, DE, USA). The bonded specimens were then stored in TESCA buffer at 4 °C for subsequent analyses using Goldner’s trichrome staining and scanning electron microscopy (SEM).

#### 2.4.1. Goldner’s Trichrome Differential Staining

For the differential staining and histological analysis, bonded dentin slabs were mounted on PMMA blocks and sectioned perpendicularly to the A/D interface into 3μm thick slices using a tungsten carbide knife mounted on a Polycut S “sledge” microtome (Leica, Wetzlar, Germany), according to Spencer’s protocol [29]. Following microtomy procedure, the remaining bonded slabs were retained for SEM preparation. Five micrometric sections were obtained from each bonded dentin slab. These sections were mounted on glass slides and stained using Goldner’s trichrome protocol [29,30]. The stained slides were dehydrated through graded ethanol and xylene series, cover-slipped using mounting media, and examined under a Nikon E800 light microscope (Nikon, Tokyo, Japan). One photomicrograph was captured per slide at 100× magnification.

#### 2.4.2. Scanning Electron Microscopy (SEM)

For SEM, the remaining bonded dentin slabs were treated with 5N hydrochloric acid for 30 s, rinsed with distilled water, and then immersed in 5% sodium hypochlorite (NaOCl) for 30 min. The specimens were further rinsed and dehydrated in ascending ethanol concentrations (50%, 70%, and 85% for 15 min each, followed by 95% and two sequential rounds of 100% ethanol for 30 min each). After overnight drying, samples were mounted on aluminum stubs and sputter-coated with ~20 nm of gold–palladium. Imaging was performed using a field-emission SEM (FEI/Philips XL30, Eindhoven, The Netherlands) at 5 kV. Five photomicrographs were acquired per specimen.

The thickness of the A/D bonding interfaces was measured directly from all photomicrographs using ImageJ software version 1.54m (ImageJ, NIH, Bethesda, MD, USA). For both Goldner’s trichrome staining and SEM analyses, the average value from each bonded dentin slab (n = 5 per group) was used for statistical analysis.

### 2.5. Microtensile Bond Strength Testing

For the microtensile bond strength (μTBS) test, the remaining twenty-four dentin slabs were randomly assigned to the experiment groups (n = 6 per group). The dentin surfaces were etched according to their respective group protocols (Table 1). Following etching and adhesive application as previously described, four layers of 3M Filtek Z250 composite (St. Paul, MN, USA) were applied to each specimen, with each layer approximately 1.5 mm thick. Each layer was light-cured using the same parameters employed for the adhesive. The restored specimens were then stored in deionized water at 37 °C overnight.

Following storage, each specimen was sectioned perpendicularly to the adhesive/dentin interface (Buehler) to obtain stick-shape specimens with an average cross-section of 0.7 × 0.7 mm and a length of 9–10 mm. The sticks from each restored specimen were initially divided into two storage solution groups: TESCA buffer or bacterial collagenase solution. Subsequently, these were further allocated to different evaluation time points: immediately (IM), 1 week (1w), 2 weeks (2w), 4 weeks (4w), 17 weeks (17w), and 1 year (1y). Specimens were stored at 37 °C, and storage solutions were replaced weekly to prevent bacterial contamination and maintain enzymatic activity.

At the designated time point, the cross-sectional area of each stick was measured using a digital caliper (Marathon, Richmond Hill, ON, Canada), and the specimens were subjected to tensile loading using a universal testing machine (Bisco, Schaumburg, IL, USA) at a crosshead speed of 1 mm/min until failure. The fracture force (N) was recorded and divided by the cross-sectional area (mm^2^) to calculate the μTBS in megapascals (MPa). Fracture modes were examined under a stereomicroscope at 40× magnification and classified into three categories based on the involved substrates: composite/adhesive (failure within the composite and adhesive), adhesive/dentin (failure at the adhesive and dentin interface), and mixed (failure involving composite, adhesive, and dentin). Additionally, five representative de-bonded specimens per group, with bond strengths closest to the group mean, were selected for scanning electron microscopy (SEM). These specimens were mounted on aluminum stubs, vacuum-dried overnight, coated with carbon, and examined under SEM at 15 kV.

### 2.6. Statistical Analysis

Data were assessed for normality and homogeneity of variance using the Kolmogorov–Smirnov and Levene’s tests, respectively. A one-way ANOVA was used to analyze data from collagenolytic activity and A/D bonding interfacial evaluations, followed by Games–Howell and Tukey post hoc tests, respectively. Microtensile bond strength data were analyzed using a three-way ANOVA, followed by Tukey’s post hoc test for multiple comparisons. Statistical significance was set at α = 0.05. All analyses were performed using IBM SPSS Statistics version 28.0.1.1 (IBM Corp., Armonk, NY, USA).

## 3. Results

### 3.1. Collagenolytic Activity of Endogenous MMPs

**Figure 2:**
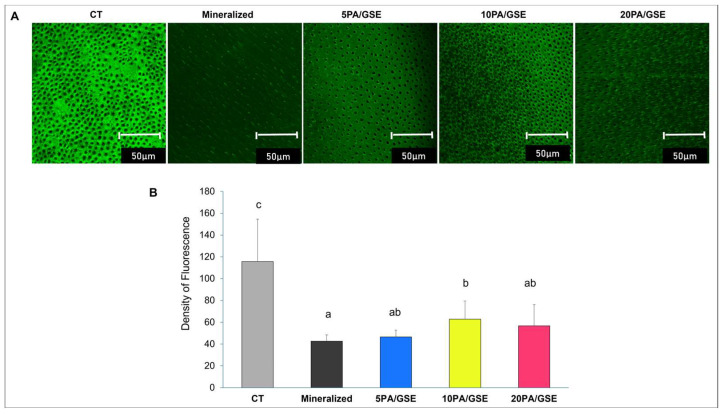
(**A**) Representative green-channel images from in situ zymography showing collagenolytic activity mediated by endogenous MMPs on mineralized dentin and dentin etched with CT and GSE-modified etchants. Green fluorescence means collagenolytic activity after 72 h incubation with fluorescein-conjugated gelatin. (**B**) A quantitative analysis of the emitted green fluorescence density indicating collagenolytic activity is shown. Statistically significant differences (*p* < 0.05) are indicated by different letters.

The results from in situ zymography are shown in Figure 2. Representative fluorescence images acquired in the green channel are displayed in Figure 2A, with a quantitative analysis in Figure 2B. A statistical analysis revealed that collagenolytic activity was significantly affected by type of acid etchant used (*p* < 0.001). Overall, all GSE-modified etchants significantly reduced collagenolytic activity compared to the control (CT) group (*p* < 0.05), regardless of acid concentration. Notably, the 5PA/GSE and 20PA/GSE groups showed collagenolytic activity levels comparable to mineralized dentin (*p* > 0.05), although not statistically different from 10PA/GSE (*p* = 0.079 and *p* = 0.944, respectively).

### 3.2. Differential Staining Technique

**Figure 3:**
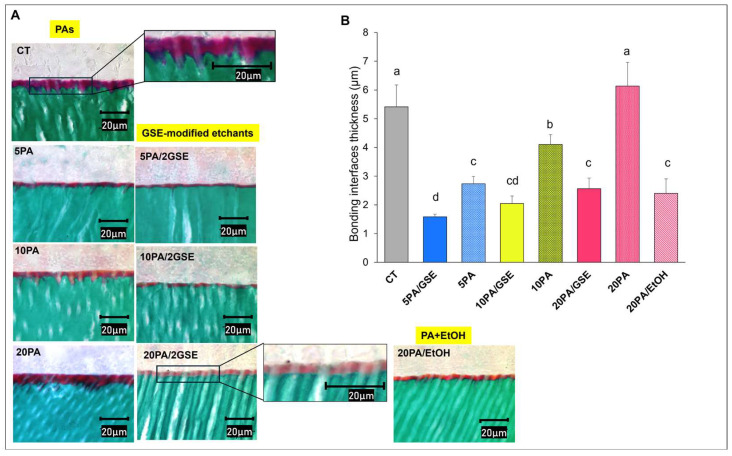
(**A**) Representative Goldner’s trichrome-stained 5 µm thick sections of the adhesive/dentin bonding interfaces, formed using control (CT, commercial 32% phosphoric acid gel), pure phosphoric acid (PA) at different concentrations, GSE-modified etchants, and 20% PA + ethanol (20PA/EtOH), displayed in columns. Green staining represents mineralized dentin, beige indicates adhesive, purple/red marks exposed collagen fibrils, and orange depicts partially encapsulated collagen fibrils by adhesive. (**B**) Quantitative analysis of interface thickness for all experimental conditions. Different letters indicate statistically significant difference (*p* < 0.05).

Light micrographs of Goldner’s trichrome-stained sections of A/D interfaces are shown in Figure 3A. In this technique, mineralized dentin collagen stains green, exposed collagen/proteins stain red or purple, partially coated collagen stains orange, and pure adhesive appears pale beige or remains unstained [29,30]. Quantitative measurements (Figure 3B) revealed that CT yielded the thickest interfaces (5.41 ± 0.55 µm), significantly great than those from all GSE-modified etchants (*p* < 0.001).

Interface thickness increased significantly with higher concentrations of unmodified phosphoric acid: 5PA (2.71 ± 0.42 μm), 10PA (4.22 ± 0.20 μm), 20PA (6.10 ± 0.60 μm) (*p* < 0.001). However, when GSE was incorporated, interface thickness was significantly reduced at all acid concentrations: 5PA/GSE (1.58 ± 0.8 µm), 10PA/GSE (2.05 ± 0.42 µm), 20PA/GSE (2.54 ± 0.17 µm) (*p* < 0.001). Interestingly, adding ethanol to 20PA (20PA/EtOH) also significantly reduced interface thickness compared to pure 20PA (*p* < 0.001), resembling the effect of 20PA/2GSE.

### 3.3. Scanning Electron Microscopy (SEM)

**Figure 4:**
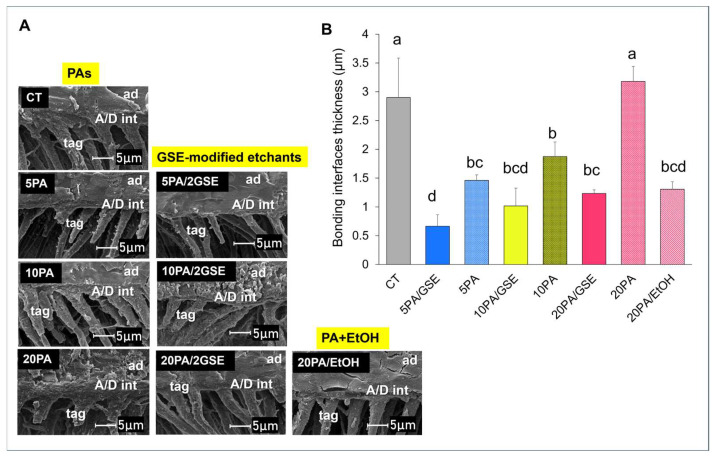
(**A**) Representative SEM micrographs of acid-bleach-treated adhesive/dentin (A/D) interfaces formed by CT, pure phosphoric acid (PA) at different concentrations, GSE-modified etchants, and 20% PA + ethanol (20PA/EtOH), displayed in columns. (**B**) Quantitative analysis of A/D interface (hybrid layer) thickness for all experimental conditions. Different letters indicate statistically significant difference (*p* < 0.05). A/D int: adhesive/dentin interface; ad: adhesive; tag: resin tag.

Representative SEM images of A/D bonding interfaces are presented in Figure 4A, with the quantitative analysis shown in Figure 4B. The results confirmed trends observed in the staining results, although overall interface thickness appeared thinner under SEM. CT produced the thickest A/D interfaces (2.90 ± 0.69 µm), significantly greater than all GSE-modified groups (*p* < 0.05), but comparable to 20PA (*p* > 0.05). Among GSE-modified etchants, the A/D interface’s thickness increased with acid concentration, though only 5PA/GSE (0.66 ± 0.20 µm) and 20PA/GSE (1.23 ± 0.06 µm) differed significantly (*p* < 0.05), while 10PA/GSE (1.01 ± 0.31 µm) was not statistically different from 5PA/GSE (*p* > 0.05).

### 3.4. Microtensile Bond Strength

**Figure 5:**
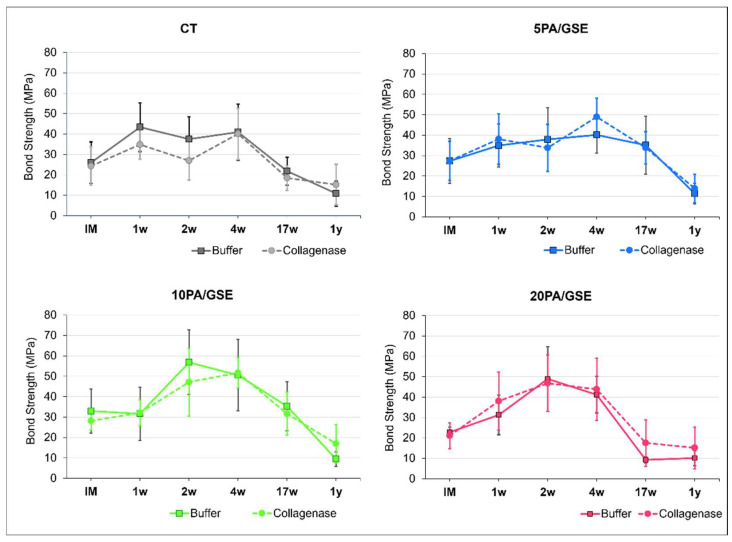
Mean and standard deviation (MPa) of microtensile bond strength for each etchant group stored in collagenase or buffer solution, evaluated over 1 year.

**Figure 6:**
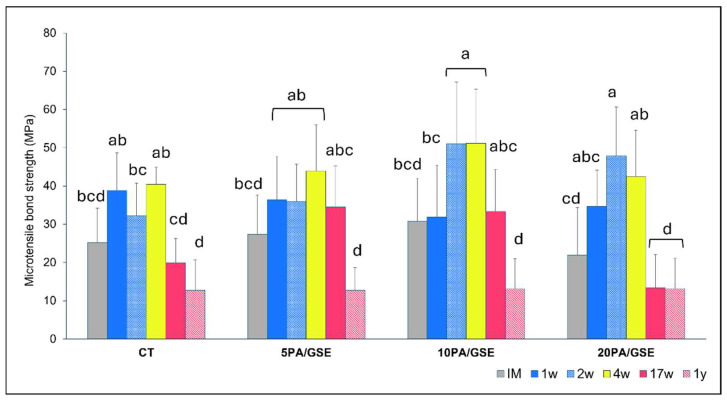
Mean and standard deviation (MPa) of microtensile bond strength showing interactions between the factors “acid etchant” and “storage time”. Consequently, this figure reflects both within-group (i.e., storage time within each etchant) and between-group (i.e., comparisons among etchants) differences. Different letters indicate statistically significant differences (*p* < 0.05).

Bond strength data are shown in Figure 5 and Figure 6. Figure 5 illustrates μTBS performance over time for each etchant group across both storage solutions. A three-way ANOVA revealed significant effects for the factors “acid etchant” (*p* = 0.002) and “time” (*p* < 0.001), as well as their cross-interaction (*p* < 0.001), while “storage solution” had no significant effect (*p* = 0.966). Figure 6 summarizes statistical analysis and pairwise comparisons over time. All groups exhibited increased bond strength after IM (immediately measured), although the magnitude and duration varied. A statistically significant increase was observed only in 10PA/GSE (at 2w and 4w vs. IM and 1w) and 20PA/GSE (at 2w and 4w vs. IM) (*p* < 0.05). Long-term degradation was observed in all groups when compared to their respective peak values. Notably, CT and 20PA/GSE showed significant reductions beginning at 17w (*p* < 0.01), whereas degradation for 5PA/GSE and 10PA/GSE became significant only after 1 year (*p* < 0.001).

**Figure 7:**
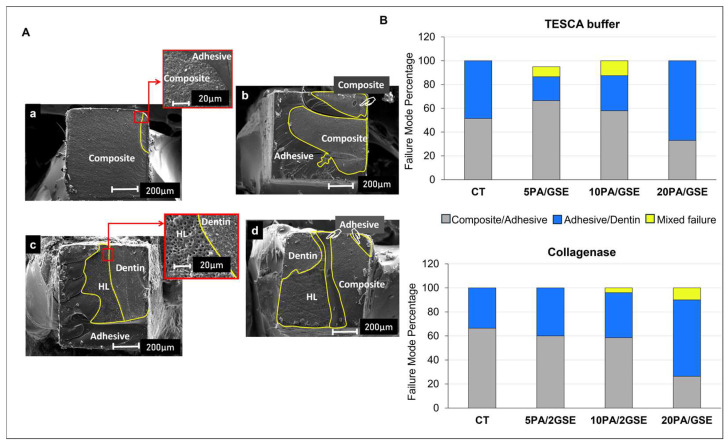
(**A**) Representative SEM micrographs illustrating fracture patterns. (**a**,**b**) Composite/adhesive fractures; (**c**) adhesive/dentin fractures; (**d**) mixed fractures. HL = hybrid layer. (**B**) The distribution of fracture patterns as percentages, determined by SEM, for de-bonded specimens from all acid etchants over 1 year, stored in TESCA buffer (top) or collagenase solution (bottom).

Representative SEM images of fracture modes are shown in Figure 7A, with distribution data in Figure 7B (averaged at 1 year for both storage solutions). A higher incidence of adhesive/dentin failures was observed in the 20PA/GSE group, regardless of storage solution (67% in TESCA buffer solution and 63.5% in collagenase solution).

## 4. Discussion

The present study is the first to demonstrate the effects of GSE-modified etchants with varying concentrations of phosphoric acid (PA) on dentin bonding interfaces and the collagenolytic activity of endogenous MMPs in etched dentin. Among the endogenous enzymes present in dentin, collagenases (MMP-8) and gelatinase A (MMP-2) are the most abundantly detected and closely associated with collagenolytic activity, followed by gelatinase B (MMP-9) [31,32] and cathepsins [33,34]. Our in situ zymography assay was designed to detect collagenase/gelatinase activity, thereby providing relevant evidence for MMP-2, -8 and -9 involvement. The findings of this study revealed that collagenolytic activity varied among different etchants, supporting our first hypothesis. In mineralized dentin, MMPs remain trapped in their proforms within the calcified matrices [27,32]. Thus, mineralized dentin served as a control in this study due to its characteristically low collagenolytic activity. The presence of apatite crystallites likely inhibits MMP access to collagen by sterically blocking their catalytic sites [35]. Acid etching removes these crystallites and disrupts the cysteine–zinc coordination within the MMP domain structure, thereby promoting activation—particularly that of MMP-2, which can subsequently activate MMP-8 and -9 [36,37]. Although activated MMPs remain stable under acidic conditions, their collagenolytic functionality requires a neutral pH, which can be obtained by dentin’s buffering capacity, saliva [32], or adhesives with a relatively higher pH than PA during the bonding process [7]. In our experiments, PBS buffering also helped achieve a neutral environment, which may explain significantly higher collagenolytic activity in the CT group (*p* < 0.05).

Previous studies have demonstrated that the proteolytic activity of endogenous MMPs is higher following treatment with 10% PA (pH~1.0) compared to 37% PA (pH~0.4) [26]. According to their rationale, acid etching with 10% PA results in the formation of dicalcium phosphate dihydrate (CaHPO_4_·2H_2_O), a precipitate with very low solubility in water. This leads to slower dissolution, and a prolonged acidic environment, which in turn promotes greater MMP activation. In contrast, higher concentrations of PA (32–37%) causes rapid demineralization and the formation of more readily dissolvable precipitates such as dibasic calcium phosphate monohydrate (Ca(HPO_4_)_2_·H_2_O), which gradually dissolve and expose collagen substrates to MMPs, thus delaying the onset of collagenolytic activity [26].

However, our findings revealed an opposite trend. The 10% PA with GSE group (10PA/GSE) exhibited nearly 50% lower collagenolytic activity compared to the CT group (32% PA, pH at ~0.1; *p* = 0.022). All GSE-modified etchants also showed significantly reduced activity (*p* < 0.05). These results indicated that lower PA concentrations did not enhance, but rather mitigated, collagenolytic activity. One explanation may lie in differences in experimental protocol. Unlike the immediate or 24 h post-demineralization measurements in DeVito-Moraes’ study, our protocol involved neutralization in PBS for 24 h, followed by a 72 h incubation in fluorescein-conjugated gelatin at physiological pH (~7.4), as recommended by the manufacturer to maximize detection sensitivity. This extended exposure to neutral conditions may have allowed more MMPs—especially those activated by acid etching—to regain enzymatic function, which more closely mimics clinical conditions where buffering from dentin, saliva, and adhesives quickly follows acid application [32]. Secondly, the lower collagenolytic activity observed with GSE etchants may be partially attributed to the milder demineralization by lower PA concentrations and the formation of less soluble precipitates, which may physically shield collagen fibrils from MMP access [38,39]. This may also explain why the 5PA/GSE group, though not statistically different, exhibited activity levels similar to mineralized dentin (*p* = 0.624), lower than the 10PA/GSE and 20PA/GSE groups. Most importantly, the collagen-protective effect of GSE etchants is likely driven by its crosslinking properties [18,20]. Proanthocyanidins (PACs) in GSE can interact with collagen and proteins through various mechanisms [22], with hydrogen bonding and hydrophobic interactions being predominant under low pH conditions, where covalent bonding is less favored [21]. This crosslinking stabilizes demineralized collagen by concealing MMP cleavage sites and limiting enzymatic access [14]. Additionally, GSE may also bind to catalytic or non-catalytic domains of MMPs, leading to molecular immobilization or irreversible conformational changes that prevent them from recognizing and cleaving collagen [40]. Together, these mechanisms explain how GSE-modified etchants protect dentin collagen against collagenolytic degradation.

We further evaluated the effects of GSE-modified etchants with varying PA concentrations on interface formation and long-term dentin bonding stability. While prior studies have only explored 10% PA [18,19], the optimal acid concentration for GSE-containing etchants to achieve effective A/D interface formation and durable bonds remains unclear. Because crosslinkers can interact with dentin minerals and potentially hinder acid diffusion and mineral removal [41], we critically assessed whether GSE affects etching efficacy. Statistical analysis revealed that all GSE-modified etchants produced thinner A/D bonding interfaces, thus supporting our second research hypothesis. Differential staining showed reductions in thickness ranging from 52.6% (20PA/GSE) to 70.64% (5PA/GSE), which SEM corroborated with reductions from 57.47% up to 77.11%, respectively. To further investigate the cause of interface thinning, we tested pure PA at the same concentrations as the GSE groups. The addition of ethanol to 20PA reduced etching depth similarly to 20PA/GSE, suggesting ethanol, rather than GSE, may impair hydrogen ion activity and thereby decrease etching efficacy.

The variation in interface thickness between the two techniques stems from differences in specimen preparation. SEM captures the cured adhesive infiltration into the collagen network, but does not reveal etched structures directly. In contrast, differential staining (Goldner’s trichrome) highlights a more comprehensive view of the A/D interface components, including dentin, adhesive, as well as exposed collagen and poorly infiltrated zones, where adhesive fails to fully encapsulate collagen fibrils, leaving them only partially protected [29,30]. As a result, it provides a more sensitive characterization of interfacial integrity. In all GSE-modified groups, thinner exposed and partially demineralized/infiltrated collagen zones (stained red, purple, or orange) were observed. Interestingly, fewer purple-stained zones were noted compared to pure PA and CT groups, suggesting improved adhesive penetration, possibly due to GSE-induced collagen crosslinking. While the shallower etching depths by GSE-modified etchants naturally result in thinner exposed collagen zones, the reduced prevalence of purple/red staining—especially upon closer examination (see image inserts)—further supports collagen receptiveness to adhesive. This may be attributed to the structural reinforcement provided by GSE crosslinking, which can stabilize the collagen network against collapse during air-drying, thus facilitating better adhesive infiltration [20]. These findings are consistent with SEM observations in both sound [41] and caries-affected dentin [18]. Importantly, although GSE-modified etchants resulted in thinner A/D interfaces, no significant difference in immediate bond strength (IM) was observed, indicating GSE-modified etchants did not compromise bonding performance, as observed by Reis et al. (2005) [42].

The bond strength results in this study demonstrated that both the type of acid etchant and the evaluation time significantly influenced outcomes, including a notable interaction effect between them. In other words, the bond strength changed over time depending on the specific etchant used, supporting only partial acceptance of the third hypothesis. When tested immediately (IM), GSE-modified etchants exhibited similar or slightly higher bond strengths compared to the commercial control (CT), although the differences were not statistically significant. This suggests that incorporating GSE into etchants did not compromise the bonding performance or adhesive polymerization efficacy. All groups showed a general increase in bond strength from IM to later time points, likely due to post-polymerization effects. This phenomenon, where unreacted double bonds in the adhesive matrix continue to polymerize in the presence of residual radicals, has been previously reported [43,44,45]. Interestingly, a statistically significant increase was observed only in the 10PA/GSE and 20PA/GSE groups after 2w compared to IM. This may be attributed to GSE-induced collagen crosslinking, which can lead to the partial dehydration of the collagen matrix [11,12,46]. A reduced water content likely facilitates monomer approximation and minimizes phase separation, thereby enhancing the polymer network formation and improving the bond strength [47,48]. In contrast, the 5PA/GSE group, which induced less demineralization and exposed less collagen, did not show a significant bond strength improvement—likely due to limited matrix modification insufficient to influence polymerization efficiency.

The aging of the A/D interface is driven by multiple interrelated mechanisms that synergistically contribute to the progressive degradation of bond strength and eventual failure of composite restorations [3,4,49,50]. This degradation process involves the breakdown of one or more components of the A/D interface, including the adhesive resin, dentin collagen matrix, and residual hydroxyapatite crystallites [3,49]. In our study, all experimental groups, including those treated with GSE-modified etchants, exhibited significant bond degradation over time. At the first glance, this may appear contradictory, given the substantial inhibition of collagenolytic activity observed in the GSE etchant groups (within ~96 h of neutralization). Collagen degradation within the hybrid layer by MMPs has indeed been associated with the gradual weakening of resin–dentin bonds [3,4,49,51]. However, these findings are not entirely unexpected. The crosslinking treatment delivered by GSE during acid etching primarily reinforces the collagen matrix and reduces MMP-mediated collagenolysis, thereby addressing only one aspect of interface degradation. Indeed, GSE treatment does not mitigate the inherent hydrophilic and acidic nature of dental adhesives, which contributes to water sorption at the interface. This promotes hydrolytic degradation of the polymer network, ultimately compromising long-term bond stability [6,49,52]. As the adhesive polymers degrade, they expose more collagen fibrils, which are then subject to mechanical fatigue and hydrolytic breakdown, and renewed collagenolytic activity from endogenous or exogenous proteases [4,49,50].

A notable positive aspect of the degradation process observed with the GSE-modified etchants is the delayed onset of significant bond deterioration. Specifically, while both the CT and 20PA/GSE group started to show a significant reduction in bond strength at 17w compared to their respective initial plateaus (*p* < 0.01), this decline occurred only after one year for the 5PA/GSE and 10PA/GSE groups (*p* < 0.001). These findings suggest that, although limited, acid etching with 5PA/GSE and 10PA/GSE contributed to greater bond stability over time, consistent with the results reported by Rey et al. [25]. The diminished long-term performance of the 20PA/GSE group is likely due to an over-etching effect, as previously speculated by Liu et al. (2014) [21] and supported by the slightly greater etching depth observed in Figure 3 and Figure 4. At higher concentrations such as 20% PA, an imbalance in the diffusion rates of PA and GSE may occur. GSE, which is rich in (epi)catechin monomers and oligomers with relatively higher molecular weights, is hydrophobic and prone to forming larger micellar structures in acidic hydroalcoholic solutions [53,54]. Consequently, its diffusion through water-filled tubules is slower than that of PA. Additionally, demineralized collagen fibers intertubular regions may further impede GSE diffusion, leaving the deeper collagen fibers at the etching front unprotected and vulnerable to degradation [21]. This probably accounts for the higher percentage of adhesive/dentin failures (Figure 7) observed in the 20PA/GSE group, indicating that the over-etched collagen became the weakest link for the A/D interface. This certainly explains the reductions in bond strength values observed by De-Paula et al. when using GSE-containing 37% PA [24].

Our findings showed that storage in a solution containing a high concentration of bacterial collagenase—a form of MMP-1 at 0.1% in TESCA (~125 U/mL), which is approximately 380 times the average concentration found in human saliva [55]—did not reduce bond strength more compared to the control buffer (*p* = 0.966). This result is notable given that collagenases are the only proteases capable of completely hydrolyzing native type I collagen, while gelatinases can further degrade its fragments [36]. In the oral conditions, collagenases like MMP-8 (collagenase-2) originate from odontoblasts within dentin or from neutrophils entering the oral cavity through gingival sulcus (MMP-1 and -8) [33,38,56]. To mimic this enzyme-rich milieu, and potentially accelerate degradation, we included bacterial collagenase (MMP-1) in the storage solution. Additionally, this same enzyme has previously induced substantial or complete biodegradation of demineralized collagen specimens [11,57]. However, bacterial collagenase differs from its mammalian counterparts by exhibiting broader substrate specificity and a larger molecular weight (~116 kDa) [58], which may hinder its ability to penetrate the narrow, water-filled spaces between collagen fibrils that remain unprotected within the hybrid layer. In contrast, demineralized dentin specimens expose the collagen matrix fully, making it more susceptible to digestion. Another factor that may explain the negligible effect of bacterial collagenase on bond strength is the composition of the adhesive system—specifically, the presence of polyalkenoic acid copolymer. This component is known to form ionic bonds with dentin [59,60], possibly shielding collagen from collagenase recognition and cleavage. A similar protective effect has been reported with the 10-MDP monomer, whose interactions with dentin collagen have been shown to reduce collagenolytic degradation [61].

We acknowledge that aside from conditions involving active inflammation—particularly acute cases, where polymorphonuclear (PMN) leukocytes and other immune cells produce large quantities of MMPs and related enzymes—the activity of collagen-degrading enzymes typically requires time to exert significant effects. While protocols employing highly exaggerated enzyme concentrations may offer an accelerated model of degradation, they fall short in replicating the complex, multifactorial clinical environment. In reality, the breakdown of A/D interfaces in composition restorations is influenced by a combination of adhesive properties, host-derived immune factors (including endogenous enzymes, PMNs, and salivary enzymes), the oral microbiome, and time [9,50]. Considering this, a potential limitation of this study is the lack of comparisons with other commercial adhesives or formulations. Additionally, future studies could evaluate A/D interfaces created with GSE-modified etchants under alternative storage conditions that better mimic human saliva or examine their impact on oral bacteria biofilms. From a translational perspective—where the microtensile bond strength of “aged” specimens has been correlated with clinical outcomes [62]—the delayed degradation observed in GSE-modified etchants, particularly with 5% and 10% PA, may represent a promising strategy to extend the durability of composite restorations.

## 5. Conclusions

Acid etching with GSE-modified etchants presents a viable strategy in bonding protocols to remarkably reduce the collagenolytic activity of endogenous MMPs in dentin without compromising the bond strength, despite thinner A/D interfaces. When combined with 5% and 10% PA, this approach offers a notable improvement in the stability of the adhesive–dentin bond, particularly by delaying the onset of bond degradation over time. However, as long-term bonding durability depends on the integrity of both the adhesive and dentin collagen matrix, stabilizing only one phase may be insufficient to ensure a lasting performance. Interestingly, more aggressive protocols, such as treatment with collagenase solution, did not accelerate bond strength degradation compared to TESCA buffer. Nevertheless, these results should be cautiously interpreted since such conditions do not accurately simulate the host oral environment involved in the breakdown of A/D interfaces.

## Figures and Tables

**Table 1:** Composition, application protocol, and pH of each acid etchant used in this study.

Group	Final Composition (Percentage by Total Weight)	Application Protocol	pH
Control (CT): Scotchbond Universal Etchant; 3M ESPE, St. Paul, MN, USA, Lot. 542642	32% phosphoric acid (approximately), water, synthetic amorphous silica, polyethylene glycol, aluminum oxide.	- Apply the acid etchant gel to the dentin; - Allow to react for 15 s; - Rinse thoroughly with deionized water for 30 s.	~0.1
5PA/GSE	5% phosphoric acid, 73% deionized water, 20% ethanol and 2% GSE.	- Apply the acid etchant to the dentin; - Allow to react for 30 s; - Rinse thoroughly with deionized water for 30 s.	~1.15
10PA/GSE	10% phosphoric acid, 68% deionized water, 20% ethanol and 2% GSE.	- Apply the acid etchant to the dentin; - Allow to react for 30 s; - Rinse thoroughly with deionized water for 30 s.	~0.85
20PA/GSE	20% phosphoric acid, 58% deionized water, 20% ethanol and 2% GSE.	- Apply the acid etchant to the dentin; - Allow to react for 30 s; - Rinse thoroughly with deionized water for 30 s.	~0.45

## Data Availability

The original contributions presented in this study are included in the article. Further inquiries can be directed to the corresponding author.
